# Augmenting existing deterioration indices with chest radiographs to predict clinical deterioration

**DOI:** 10.1371/journal.pone.0263922

**Published:** 2022-02-15

**Authors:** Emily Mu, Sarah Jabbour, Adrian V. Dalca, John Guttag, Jenna Wiens, Michael W. Sjoding

**Affiliations:** 1 Department of Computer Science and Electrical Engineering, Massachusetts Institute of Technology, Cambridge, MA, United States of America; 2 Division of Computer Science and Engineering, University of Michigan College of Engineering, Ann Arbor, MI, United States of America; 3 Martinos Center, Massachusetts General Hospital, Harvard Medical School, Boston, MA, United States of America; 4 Institute for Healthcare Policy and Innovation, University of Michigan, Ann Arbor, MI, United States of America; 5 Department of Internal Medicine, University of Michigan Medical School, Ann Arbor, MI, United States of America; School of Digestive & Liver Diseases, Institute of Post Graduate Medical Education & Research, INDIA

## Abstract

**Importance:**

When hospitals are at capacity, accurate deterioration indices could help identify low-risk patients as potential candidates for home care programs and alleviate hospital strain. To date, many existing deterioration indices are based entirely on structured data from the electronic health record (EHR) and ignore potentially useful information from other sources.

**Objective:**

To improve the accuracy of existing deterioration indices by incorporating unstructured imaging data from chest radiographs.

**Design, setting, and participants:**

Machine learning models were trained to predict deterioration of patients hospitalized with acute dyspnea using existing deterioration index scores and chest radiographs. Models were trained on hospitalized patients without coronavirus disease 2019 (COVID-19) and then subsequently tested on patients with COVID-19 between January 2020 and December 2020 at a single tertiary care center who had at least one radiograph taken within 48 hours of hospital admission.

**Main outcomes and measures:**

Patient deterioration was defined as the need for invasive or non-invasive mechanical ventilation, heated high flow nasal cannula, IV vasopressor administration or in-hospital mortality at any time following admission. The EPIC deterioration index was augmented with unstructured data from chest radiographs to predict risk of deterioration. We compared discriminative performance of the models with and without incorporating chest radiographs using area under the receiver operating curve (AUROC), focusing on comparing the fraction and total patients identified as low risk at different negative predictive values (NPV).

**Results:**

Data from 6278 hospitalizations were analyzed, including 5562 hospitalizations without COVID-19 (training cohort) and 716 with COVID-19 (216 in validation, 500 in held-out test cohort). At a NPV of 0.95, the best-performing image-augmented deterioration index identified 49 more (9.8%) individuals as low-risk compared to the deterioration index based on clinical data alone in the first 48 hours of admission. At a NPV of 0.9, the EPIC image-augmented deterioration index identified 26 more individuals (5.2%) as low-risk compared to the deterioration index based on clinical data alone in the first 48 hours of admission.

**Conclusion and relevance:**

Augmenting existing deterioration indices with chest radiographs results in better identification of low-risk patients. The model augmentation strategy could be used in the future to incorporate other forms of unstructured data into existing disease models.

## Introduction

An essential characteristic of risk models used in medical decision-making is predictive performance, and new strategies should therefore prioritize the inclusion of all available data that could improve this accuracy. However, the vast majority of risk models analyze only structured data from the electronic health record (EHR) when making predictions [1–7]. In contrast, clinicians often synthesize information from multiple sources, such as radiological imaging and findings, laboratory test results, and clinical observations, when making clinical decisions [8]. Moreover, when radiologists are not given access to essential clinical information, their diagnostic decisions are negatively impacted [9, 10]. Because chest radiology data may contain critical information about a patient’s risk of deterioration in COVID-19 [11, 12], they are often used by clinicians. Augmenting existing deterioration indices with additional features extracted from chest radiographs might therefore have a significant impact on their ability to differentiate low and high risk patients.

Large influxes of hospitalized patients during the COVID-19 pandemic caused significant and acute strains on hospital resources around the globe [13]. While in-hospital deterioration models have been largely designed to identify patients at high risk for acute in-hospital complications [2, 3], one potential novel application of these indices is identifying subsets of patients at low-risk of severe outcomes for safe, early discharge to alleviate hospital strain. For example, a recent validation study of the EPIC deterioration index found that it had promising predictive performance for identifying subsets of high- and low- risk patients with COVID-19 [4, 5].

In this work, we describe a method using machine learning to augment existing deterioration indices with chest radiographs. We apply the approach to predict deterioration of patients hospitalized with COVID19, augmenting two existing EHR-based deterioration indices [4, 5]. While focusing on the clinical problem of COVID-19, this approach has broader applicability to other risk models that could be augmented with imaging data.

## Methods

This retrospective cohort study was approved by the Institutional Review Board of the University of Michigan Medical School (HUM00179831: Prediction of complications and outcomes in COVID-19 patients at Michigan Medicine). Given the retrospective nature of the data analysis, the Michigan Medicine IRB waived the requirement for informed consent among study subjects, and data were anonymized prior to analysis.

All data analysis were performed using Python.

### Study population and clinical outcomes analyzed

Because of the limited number of COVID-19 patients available for model development and testing, we used COVID-19 PCR negative patients admitted through the emergency department at Michigan Medicine (MM) and required any type of respiratory support during their hospitalization from January 1, 2020 to December 31, 2020 for model training. We used COVID-19 PCR positive patients for validation and testing during the same time period. Patients were included if they required supplemental oxygen during their hospitalization and had at least one chest radiograph performed during the first 48 hours. Patients that experienced clinical deterioration or discharge within four hours of presentation were excluded. Clinical deterioration was defined as death or the need for ICU-level therapies including invasive or non-invasive mechanical ventilation, heated high flow nasal cannula, or vasopressor support. Patients that experienced clinical deterioration prior to their first radiograph being taken were also excluded.

The analysis focused on two related clinical problems: (1) identifying low-risk patients unlikely to deteriorate who may be safe for early discharge; (2) identifying high-risk patients likely to deteriorate and need intensive care level therapies. We determined whether patients experienced a clinical deterioration event during their hospitalization using data recorded in the patient’s electronic health records. If a patient experienced two or more deterioration events during the hospitalization, we define the deterioration time to be the time of the first event.

### EHR deterioration indices

In the analysis, we augmented two different existing EHR-based deterioration indices: the Epic Deterioration Index (EDI) and MCURES [4, 5].

#### EDI

The Epic Deterioration Index (EDI) model is a proprietary prediction model used in at least 100 different hospital systems across the United States [5]. This model has been found to have fair performance and makes patient risk predictions at 15-minute intervals [5]. To compare this model with the image-augmented indices, we selected the maximum risk score for all 15-minute intervals in each four-hour interval.

#### MCURES

The MCURES index is a deterioration index locally developed at the University of Michigan. It makes predictions every four hours from 1 am to 9 pm each day, using 8 curated EHR variables: age, respiratory rate, oxygen saturation (SpO2), O2 flow rate, pulse oximetry type (intermittent or continuous), head of bed position, blood pressure (BP) patient position, pH venous blood gas (VBG) and pCO2 arterial blood gas (ABG).

### Image-augmented deterioration model

**Fig 1:**
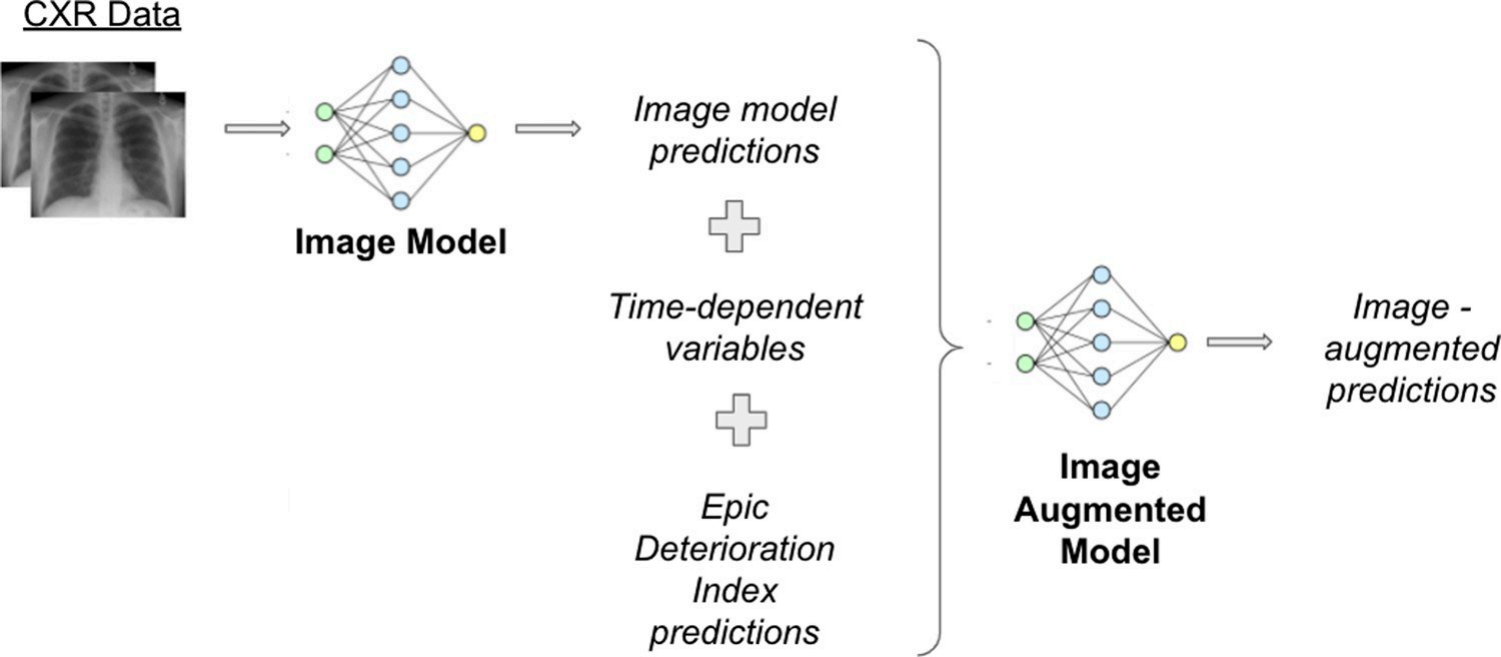
Image-augmented deterioration model. The image-augmented deterioration index combines outputs from an image model, an EHR deterioration index, and two time dependent variables encoding the time since the chest x-ray was performed and the time since the patient was admitted. Both the EHR deterioration index and image model can be varied. We provide additional details of chest x-ray preprocessing in the S1 Fig.

To augment these existing EHR deterioration indices with imaging data, we first trained an image model to predict a patient’s risk of deterioration based on the chest x-ray data alone. Then, we combined the image model outputs and deterioration index outputs as described below and illustrated in Fig 1.

To train the image model, we used a deep neural network with a DenseNet-121 architecture [14] which was first pre-trained on the CheXpert [15] and MIMIC-DICOM [16]. datasets. For image model pre-training, we pretrained a DenseNet-121 architecture with random initialization, learning rate 1e-4, and batch size of 16 on the combined MIMIC/CheXpert datasets. Data augmentation included random rotations of images between 15 and -15 degrees. We pretrained for 100 epochs and saved the model checkpoint with the best validation accuracy over these epochs.

Model hyperparameters (e.g. learning rate and training epochs) were selected based on the lowest binary cross entropy loss on the validation data.

Using a multi-task learning approach similar to Sriram et al., the image-model was fine-tuned to analyze chest x-rays and predict the likelihood of clinical deterioration over multiple time horizons (12, 24, 48, 72, 96, 120 hours, or at any point) on the COVID-19 negative patient population [17].

We combine the image model outputs, deterioration index scores and time-dependent variables to construct vector inputs to our image-augmented model. The image-augmented model consists of a feed-forward neural network with a single hidden layer of five nodes and a single-prediction output. We trained this model with a learning rate of 0.001 with binary cross-entropy loss.

**Fig 2:**
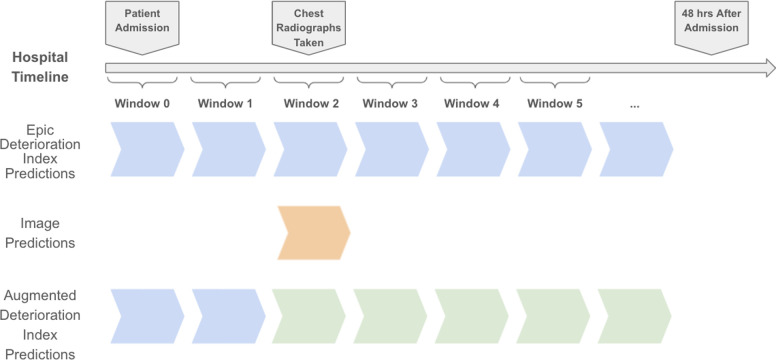
Prediction timeline during a hospitalization. A hospitalization timeline is shown for a hypothetical patient illustrating typical data availability and when predictions can occur. Image model predictions are made only during the time at which image studies are taken. The image-augmented deterioration index makes predictions for all windows after the image has been taken, using the deterioration index prediction prior to the first radiograph.

To construct the image-augmented deterioration model, the image-model output and deterioration index output were combined with two time-related variables described below. As illustrated in **Fig 2**, the EHR deterioration indices make predictions throughout the hospitalization while the image model makes predictions when chest x-rays are performed. The combined index makes predictions following the time of the chest x-ray, using the deterioration index prediction prior. At subsequent time intervals, the most recent image-model outputs are combined with the EHR risk index from the current interval. Two additional time-related variables are included encoding (i) time since the chest x-ray was performed and (ii) the time since the patient was admitted. The image model output, EHR deterioration index output, and time-related variables are then passed through a fully connected feed-forward neural network to estimate the likelihood of clinical deterioration.

#### Image-augmented deterioration model training

Model training was performed on the COVID-19-negative patient population with acute dyspnea, and the model was validated on a randomly chosen subset of 30% the COVID-19 positive population, with model hyperparameters selected based on validation performance (see S1 Fig for details). The model designed to identify low-risk patients was trained separately from a model designed to identify high-risk patients. For identifying low-risk patients, we trained the model to predict if a patient would ever deteriorate during the hospitalization. Predictions were made every four-hours starting after the patient’s chest radiograph was taken for the first 48 hours of the hospitalization. For identifying high-risk patients, we trained a separate model to predict if a patient was likely to deteriorate within the first five days of the hospitalizations. Predictions were made every four hours after the first chest x-ray during the five days of admission or up until the 4 hour interval prior to the clinical deterioration rate.

### Model evaluation

Evaluation of the EHR risk models, the image-model, and the image-augmented deterioration models was performed on COVID-19 positive patients. When evaluating how well models discriminate low-risk patients at 48 hours of observation, we calculated a patient total risk score using the mean of all predicted risk scores over the first 48 hours. Patients who met the outcome in the first 48 hours for this task were excluded. We then sorted patients by ascending risk score. We swept the model threshold for identifying low risk patients, thereby increasing the fraction of patients identified as low risk as we raised the model threshold for risk score. For each threshold, we compute the model negative predictive values (NPV) and the fraction of patients identified as low-risk.

For the Epic Deterioration Index, MCURES, and the augmented versions of each model, and the image-model alone, we determined the number of low-risk patients that each model could identify while maintaining NPVs of 95% or 90%. We also determined the fraction of patients that would be identified as low risk at these NPV thresholds if we make predictions at 24, 32, 40 and 48 hours after hospital admission. Empirical 95% confidence intervals (CIs) were calculated by bootstrapping the test set 1000 times.

When evaluating how well models could discriminate high-risk patients at risk for deterioration, the primary metric of evaluation was the area under the receiver operator characteristics curve (AUROC). We calculated the AUROC by comparing the risk scores returned by the deterioration index to the binary label of whether a patient deteriorated within the first five days for the entire patient population. The risk score is computed as the maximum of all predicted risk scores prior to the deterioration event or within the first five days.

## Results

Data from 6278 patient hospitalizations, representing 5063 unique patients, were analyzed. Training data included 5562 hospitalizations among patients without COVID-19; validation and test data included 216 and 500 hospitalizations among patients with COVID-19 (Table 1). The cohorts also included 11,496 chest x-ray images used in model training.

**Table 1:** Demographic and clinical characteristics of the patients analyzed.

Characteristic	Non-COVID-19	COVID-19
	Train Cohort	Test + Valid Cohort
Number of patients	4575	675
Number of hospitalizations	5562	716
Age. median [IQR]	64.0 [53.0, 74.0]	64.0 [53.0, 75.0]
Age Group		
[18, 25]	154 (2.8%)	12 (1.7%)
[25, 45]	675 (12.1%)	86 (12.0%)
[45, 65]	2107 (37.9%)	282 (39.4%)
[65, 85]	2295 (41.3%)	277 (38.7%)
>85	403 (7.2%)	65 (9.0%)
% Female	44.7%	42.5%
Race		
Caucasian	4447 (80.0%)	456 (63.7%)
African American	750 (13.5%)	163 (22.8%)
Other/Unknown	331 (6.5%)	83 (13.5%)
Clinical Deterioration during the hospitalization	979 (17.6%)	218 (30.4%)
Clinical Deterioration during the first 5 hospital days	663 (11.9%)	176 (24.6%)
Clinical Deterioration Reason		
Death	116 (2.1%)	9 (1.3%)
MV	255 (4.6%)	21 (2.9%)
HHFNC	412 (7.4%)	179 (25.0%)
IV pressors	196 (3.5%)	9 (1.3%)

Clinical deterioration was defined as death or the need for ICU-level therapies including invasive or non-invasive mechanical ventilation, heated high flow nasal cannula, or vasopressor support.

The median age of the COVID-19 patients was 64.0 (IQR: 53.0–74.0) years; 43.0% were female and 58.6% were white. Demographics between the non-COVID-19 and COVID-19 patients were largely similar with a few exceptions. Patients with COVID-19 were more often Black compared to non-COVID-19 patients (27.4% versus 13.5%) and deteriorate more often compared to our non-COVID-19 training cohort (31.4% vs 17.6%) (Table 1).

### Identifying low-risk patients

**Fig 3:**
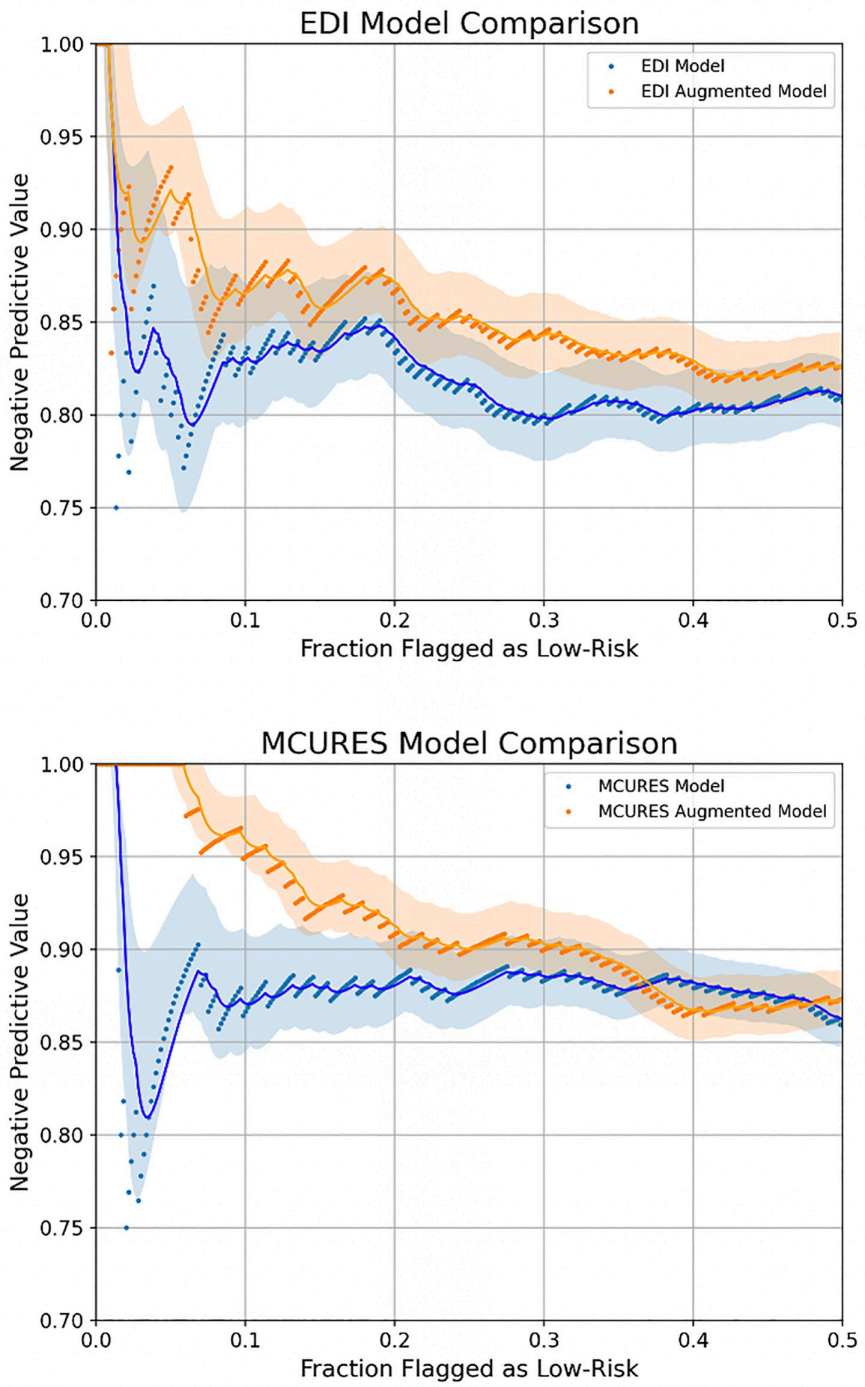
Model predictions for COVID19 positive patients within the first 48 hours of admission, shown with exponential weight moving average and 95% CIs. Each plot shows the number of patients flagged as low-risk by lowest aggregated prediction and the resulting accuracy for that fraction of patients. The top plot compares the EDI augmented model to the EDI model. The bottom plot compares the MCURES augmented model to the MCURES model.

The image-augmented EDI model and image-augmented MCURES model consistently maintained a higher negative predictive value than the non-image augmented versions as the fraction of patients considered low risk was increased (Fig 3). Models based on the Epic DI score had lower negative predictive values overall; the image augmented version had better performance than the non-augmented version. At a negative predictive value of 90%, the image-augmented EDI index identified 6.1% of patients as low risk while the non-augmented version identified 0.7% of patients as low risk. At a negative predictive value of 90%, the image-only model identifies fewer than 0.5% of patients as low-risk. The image-augmented MCURES model could identify 6.7% of the patients as low risk while maintaining a negative predictive value of 100%. At a negative predictive value of 95%, the augmented version of the MCURES model identified 11.1% of the patients as low risk while the non-augmented version identified 1.6% of the patients as low risk.

**Fig 4:**
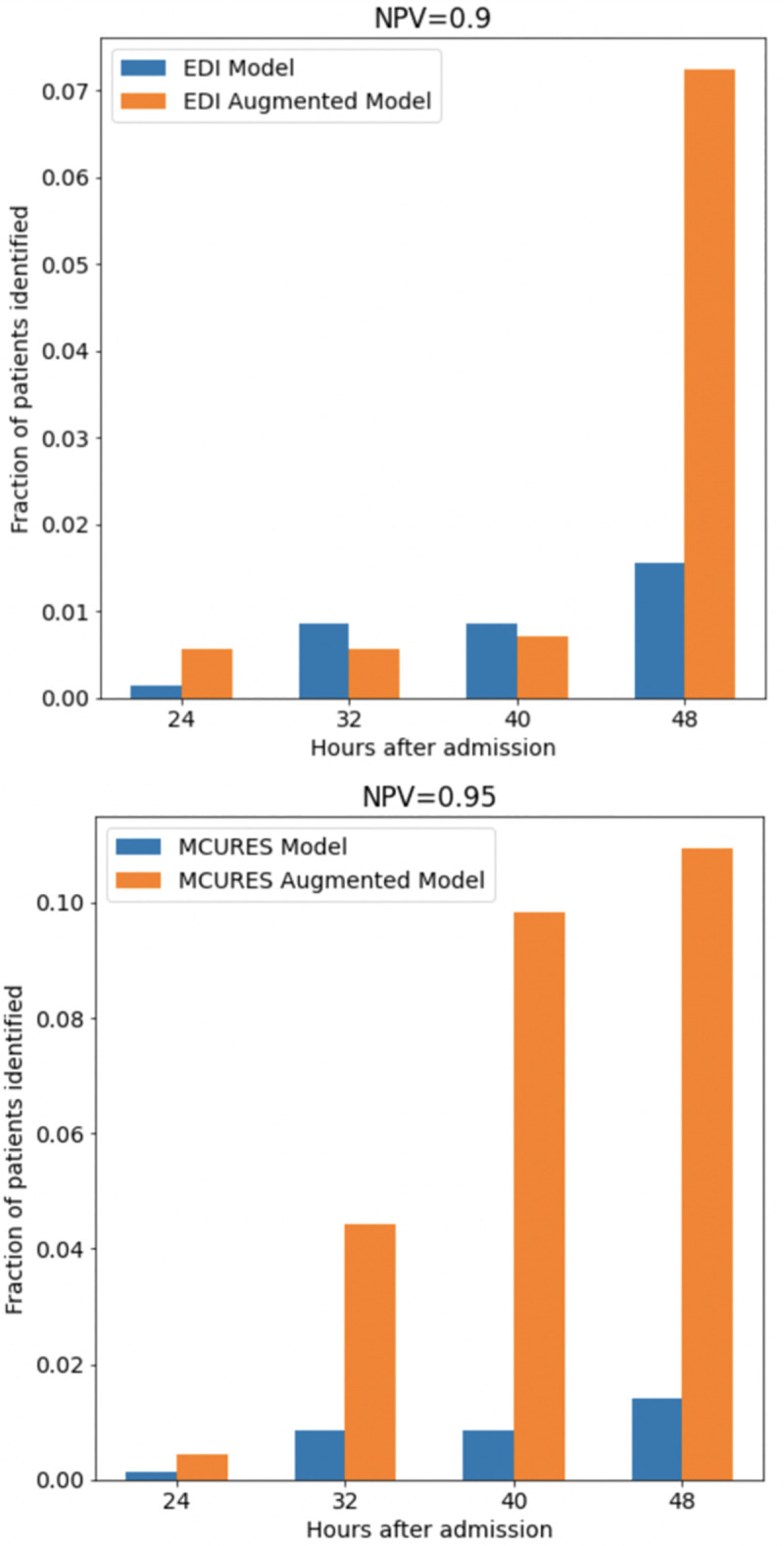
The fraction of patients correctly identified by each of the models as low-risk, shown over hours after hospital admission. Note that the MCURES and MCURES-augmented models generally have better performance over the EDI and EDI-augmented models and so we select 0.9 NPV as an appropriate cutoff for EDI and 0.95 NPV for MCURES.

We also evaluated how many patients each model could identify as low risk from hour 24 through 48 at specific negative predictive values. For this analysis, we analyzed the Epic DI model at a negative predictive value of 90% and the MCURES models at a negative predictive value at 95%. We used a lower threshold for the Epic DI model because it was unable to maintain a negative predictive value above 95%. At a NPV of 0.9, the image-augmented EDI index identified 26 more individuals out of 500 compared to the EDI model as low-risk of deterioration in the first 48 hours of admission. At a NPV and 0.95, the image-augmented MCURES index identified 48 more individuals compared to the MCURES model as low-risk of deterioration in the first 48 hours of admission. At 24, 32, and 40 hours, at a NPV of 0.9, the image-augmented EDI model performs similarly to the EDI model, but outperforms the EDI model at 48 hours (Fig 4). Across all time points, at a NPV of 0.95, the image-augmented MCURES index was able to identify a larger number of patients as low-risk of deterioration compared to the MCURES and image models alone (Fig 4).

### Identifying high-risk patients

Augmentation of the EPIC DI score yielded some improvement in the detection of high-risk patients but there was not meaningfully improvement for the MCURES index. The image-augmented Epic DI score outperformed the Epic DI score alone (0.659 [95% CI: 0.603–0.712] vs. 0.631 [95% CI: 0.571–0.686]) (Table 2). At a sensitivity of 0.8, the image-augmented Epic DI score had a specificity of 0.58 and the Epic DI model alone had a specificity of 0.54. The AUROC of the image model alone was 0.649 [95% CI: 0.593–0.699]. The augmented version of the MCURES index had an AUROC = 0.806 (95% CI: 0.761–0.847) while the non-augmented version had an AUROC = 0.800 (95% CI: 0.751–0.845) for identifying high-risk patients during the first five days of hospitalization. At a sensitivity of 0.8, the image-augmented MCURES index had a specificity of 0.77 and the MCURES index alone had a specificity of 0.76.

**Table 2:** Reported area under the receiver operating characteristic (AUROC) and 95% CIs for each deterioration index for identifying high-risk patients within the first five days of admission.

Model	Image Model AUROC	Deterioration Index AUROC	Image-Augmented Deterioration Index AUROC
Epic Deterioration Index	0.649 (0.593–0.699)	0.631 (0.571–0.686)	0.659 (0.603–0.712)
MCURES	0.649 (0.593–0.699)	0.800 (0.751–0.845)	0.806 (0.761–0.847)

## Discussion

We describe a new approach for augmenting EHR-based deterioration models with imaging data. We applied the approach to two EHR-based deterioration indices used for COVID-19, EDI and a locally-developed model called MCURES [4, 5]. When applied to data from a large hospital, the image-augmented deterioration indices identified over five times more low-risk COVID-19 patients than the deterioration indices based on EHR data alone. This approach is novel in three respects. First, the analysis is one of the first to combine EHR-based deterioration indices with chest radiographs. Second, we augmented existing models without needing to retrain these models. Third, the method generalizes well: training was performed on patients without COVID-19, but performed well in patients with COVID-19.

Automated risk indices based on clinical data recorded in the EHR are becoming increasingly common in hospital-based setting, including proprietary risk models (e.g. EDI, Rothman Index) or disease-specific or institution-specific indices (e.g. PICTURE) [5, 18]. To date, few have described how to combine these indices with new data types such as chest radiographs. Physician-based prognosis includes the synthesis of information from multiple data sources such as structured EHR data, radiological imaging or clinical observations [9, 10]. Combining these sources of information from scratch is difficult because of the differing structure and varying sampling frequency of different data sources. In this work, we described a simply approach for synthesizing information, allowing developers to augment existing work in risk prediction and disease prognosis by combining additional data-sources (e.g. chest radiographs) without having to retrain existing models.

Augmenting an already validated risk index, rather than training a new model has additional benefits. Augmentation, rather than re-training, requires less data because the augmentation model is trained on the deterioration index output (rather than its inputs), resulting in a smaller feature set during training. When using a large variety of different data sources, multimodal models trained from scratch tend to overfit to limited data [19]. By using only the outputs of existing validated models, we limit the number of features used in the image-augmented deterioration indices, mitigating the risk of overfitting.

While our goal was to develop an augmented deterioration index for patients with COVID-19, we trained the augmented models using patients who tested negative for COVID-19. One key limitation of existing machine learning models for the diagnosis and prognosis of COVID-19 is that, as with any emerging disease, a limited amount of data is available for training [11, 12, 20–22]. Because the cohorts were similar, we were able to train a model on patients who tested negative for COVID-19 but performed well on COVID-19 patients, despite our limited access to COVID-19 data. This approach may work well in other settings where there is limited access to the disease of interest, such as future emerging infectious diseases or uncommon diseases.

Augmenting validated risk models could be extended to other types of data (e.g., free-text medical histories or genomic data). While the combined model does not have to be trained from scratch, some form of additional pre-processing and training is required. For example, text data may need to be first processed through an existing language model, prior to additional training on the joint predictions of the underlying models [23].

We also found that augmentation was more effective for the EDI risk index than the local MCURES model, likely because of the significantly higher performance of the MCURES model. This suggests that augmenting global models with institution specific imaging data may help more than augmenting institution specific models.

In addition, our combined models were more useful for identifying low risk patients than for identifying high risk patients. We speculate that this is because chest radiographs may not be as helpful in constructing features for identifying high-risk patients as for identifying low-risk patients. The augmented deterioration indices improve upon the existing EHR-based indices and image model for identifying both low-risk patients for COVID-19 deterioration. This suggests that there does exist complementary information between the two modalities for identifying low-risk patients. Future work to further understand the relative value of various data modalities considered for inclusion in multimodal models is warranted.

While our models perform well on our COVID-19 test cohort, there are several limitations to our analysis. While using non-COVID-19 patients provided access to more patients for model training, we might not have identified COVID-19-specific features during the training process [24]. As increasing amounts of COVID-19 data become more available, new models could be trained using such data. Moreover, as more data becomes available, we may be able to learn to combine features of individual modalities that might give insight into how different data sources complement one another which is an exciting direction for future work. Finally, we only validated this approach on data from a single institution. Future work should include testing this approach on data across institutions.

Although risk stratification models are used frequently elsewhere in medicine, models may not utilize all sources of readily available data types for patients on which they are applied. For example, in the setting of detecting cancer in pulmonary nodules [25], validated models have been developed to determine if CT-detected lung nodules contain cancer. These models include predictors from structured data, e.g. age, sex, nodule size, nodule location, and family history [25]. While these models achieve good discrimination and calibration, incorporating images of the nodules themselves as a predictor might improve these models further [11]. This approach could potentially be applied to augment such models to improve risk stratification prediction and resulting patient care.

## Conclusion

In summary, we describe a new approach to augment existing deterioration indices with chest radiograph data. Applied to two existing models to determine risk for COVID-19 patients, this approach more accurately identified a larger fraction of patients as low-risk. We found that the image-augmented EDI was also able to improve upon performance at identifying high-risk patients.

## Supporting information

S1 Fig Image model predictions. Image model predictions for COVID19 positive patients within the first 48 hours of admission, shown with exponential weight moving average and 95% CIs. This plot shows the number of patients flagged as low-risk by lowest aggregated prediction and the resulting accuracy for that fraction of patients for the image model alone.
(TIF)

S1 File Supporting information file. (DOCX)
