# Supplementary figures and images for: Augmenting existing deterioration indices with chest radiographs to predict clinical deterioration
Source: PLoS One. 2022 Feb 15;17(2):e0263922. doi: 10.1371/journal.pone.0263922 (PMC8846502; doi:10.1371/journal.pone.0263922)

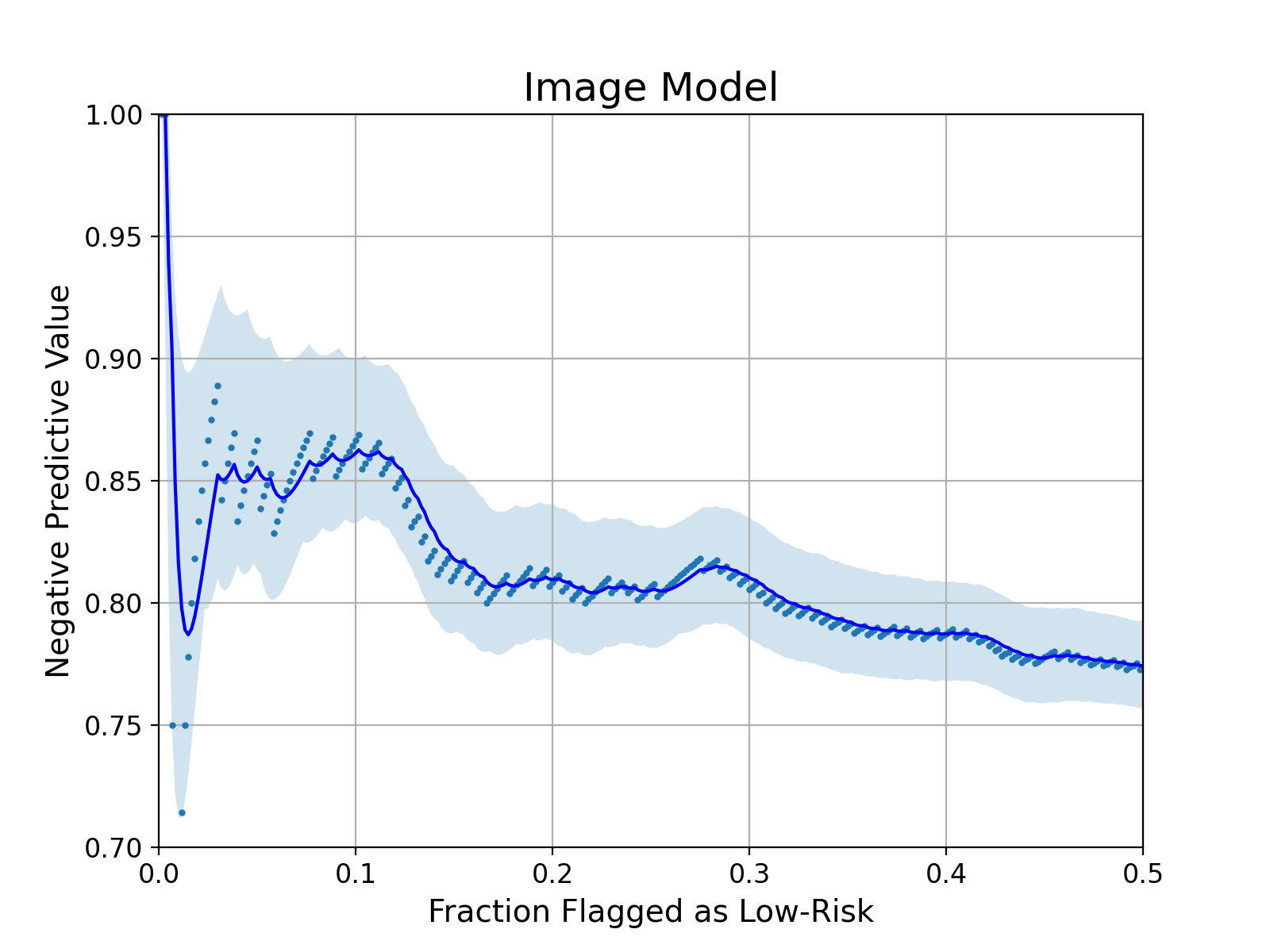

Supplement: S1 Fig — Image model predictions for COVID19 positive patients within the first 48 hours of admission, shown with exponential weight moving average and 95% CIs. This plot shows the number of patients flagged as low-risk by lowest aggregated prediction and the resulting accuracy for that fraction of patients for the image model alone. (TIF) [file pone.0263922.s001.tif]
