# Supplementary material for: Augmenting existing deterioration indices with chest radiographs to predict clinical deterioration
Source: PLoS One. 2022 Feb 15;17(2):e0263922. doi: 10.1371/journal.pone.0263922 (PMC8846502; doi:10.1371/journal.pone.0263922)
Supplement: S1 File — (DOCX) [file pone.0263922.s002.docx]

**Supporting information**

**S1 Table.** Demographic and Clinical Characteristics.

| **Characteristic** | **Low-Risk Patient Cohort** | **High-Risk Patient**  **Cohort** |
| --- | --- | --- |
| Number of patients | 420 | 488 |
| Number of hospitalizations | 428 | 500 |
| Age. median [IQR] | 64.0 [52.0, 75.0] | 64.0 [53.0, 75.0] |
| Age Group  [18, 25]  (25, 45]  (45, 65]  (65, 85]  >85 | 6 (1.4%)  54 (12.6%)  164 (38.3%)  170 (39.7%)  38   (8.9%) | 6   (1.2%)  60 (12.0%)  197 (39.4%)  197 (39.4%)  44   (8.8%) |
| % Female | 44.2% | 43.0% |
| Race  White  Black  Other/Unknown | 251 (58.6%)  114 (26.6%)  52   (14.8%) | 293 (58.6%)  137 (27.4%)  58  (14.0%) |
| Clinical Deterioration during the hospitalization  Clinical Deterioration during the first 5 hospital days | 85 (19.9%)  49 (11.4%) | 157 (31.4%)  121 (24.2%) |
| Clinical Deterioration Reason  Death  MV  HHFNC  IV pressors | 7 (1.6%)  6 (1.4%)  68 (15.9%)  4 (0.9%) | 8 (1.6%)  17   (3.4%)  126 (25.2%)  6   (1.2%) |

^a^ Clinical deterioration was defined as death or the need for ICU-level therapies including invasive or non-invasive mechanical ventilation, heated high flow nasal cannula, or vasopressor support.

^b^ Mechanical ventilation

^c^ High-heated flow nasal cannula

^d^ Vasopressor support

**Image Model Details**

**Pretraining on Public Chest Radiograph Datasets**

We pretrain our CXR model on the preprocessed CheXpert and MIMIC-CXR-JPG images. The CheXpert cohort was retrospectively collected from the Stanford Hospital between October 2002 and consists of 224,316 chest radiographs from 65,240 patients. The MIMIC-CXR dataset was retrospectively collected from the Beth Israel Deaconess Medical Center in Boston, MA and consists of 377,110 chest radiographs from 227,835 studies. Image labels from the CheXpert dataset included fourteen conditions including atelectasis, cardiomegaly, edema, and pneumonia.

**Chest Radiograph Preprocessing**

Chest radiographs taken during hospitalization were obtained in the form of digital imaging and communications in medicine (DICOM) files. Global histogram equalization was first applied to the images to increase contrast in the original images. Then, images were resized to 512 x 512 pixels, while preserving aspect ratio. Each patient had a study taken during hospitalization, which is a collection of radiographs taken at the same point in time. We only considered frontal images within a study, and excluded all radiographs that were taken after a patient met the criteria for clinical deterioration needs. To compute a prediction per study, we averaged over all radiographs in a particular study if multiple were taken.

**Image Augmented Model Calibration Details**

For windows prior to when a radiograph was taken, we use the EDI score for the window prediction. For windows after a radiograph was taken, we trained a network to compute an image-augmented EDI using both the radiograph and the EDI score. To aggregate a patient risk score from both the EDI model and the image-augmented model, we calibrated each model using the validation set. For each model, we divided the model predictions into quintiles and plotted the average prediction over the true probability of deterioration for each quintile. We then computed a line of best fit for each model to map each model’s predictions to calibrated predictions. We then used the new calibrated predictions to aggregate over all windows. Image model predictions perform worse at predicting low-risk patients in comparison to EHR or augmented model predictions (Figure S1).
